# General Perception of Doctor–Patient Relationship From Patients During the COVID-19 Pandemic in China: A Cross-Sectional Study

**DOI:** 10.3389/fpubh.2021.646486

**Published:** 2021-07-06

**Authors:** Yanan Zhou, Shubao Chen, Yanhui Liao, Qiuxia Wu, Yuejiao Ma, Dongfang Wang, Xuyi Wang, Manyun Li, Yunfei Wang, Yingying Wang, Yueheng Liu, Tieqiao Liu, Winson Fu Zun Yang

**Affiliations:** ^1^Department of Psychiatry, National Clinical Research Center for Mental Disorders, The Second Xiangya Hospital of Central South University, Changsha, China; ^2^Department of Psychiatry, Hunan Brain Hospital, Changsha, China; ^3^Department of Psychiatry, Sir Run Run Shaw Hospital, Hangzhou, China; ^4^Department of Psychiatry and Psychotherapy, University Hospital Rechts der Isar, Technical University of Munich, Munich, Germany; ^5^Department of Psychological Sciences, College of Arts & Sciences, Texas Tech University, Lubbock, TX, United States

**Keywords:** COVID-19, doctor-patient relationship, trust, communication, medical violence

## Abstract

The doctor–patient relationship (DPR) is essential in the process of medical consultations and treatments. Poor DPR may lead to poor medical outcomes, medical violence against doctors, and a negative perception of the healthcare system. Little is known about how DPR is affected during this novel coronavirus disease 2019 (COVID-19) pandemic. This cross-sectional study aimed to explore the DPR during the COVID-19 pandemic. There were 1,903 participants in China (95% response rate) who were recruited during the pandemic online *via* convenience and snowball sampling. Several questionnaires were used to evaluate participants' attitudes toward DPR, including the Patient–Doctor Relationship Questionnaire (PDRQ-9), Chinese Wake Forest Physician Trust Scale (C-WFPTS), a survey on medical violence against doctors, factors that affect and improve DPR, and general trust in medical services. Results revealed that DPR improved, and doctor–patient trust increased compared to participants' retrospective attitude before the pandemic. In addition, patients' violence against doctors decreased during the pandemic. Better doctor–patient trust and lower violence toward doctors are related to better DPR. Furthermore, we found that the main factors that could improve DPR include communication between doctors and patients, medical technology and services, and medical knowledge for patients. This study helped to better understand DPR in China, which may contribute to future health policies and medical practices in order to improve DPR and doctor–patient trust.

## Introduction

The novel coronavirus disease 2019 (COVID-19) is an emergency respiratory disease caused by a novel coronavirus. COVID-19 is highly infectious, and the symptoms include fever, dry cough, fatigue, dyspnea, and others (1). In China, many major cities were locked down gradually since January 2020, and the whole country was locked down eventually. Many medical workers either worked in the frontline locally or have been sent to Hubei Province as there was a shortage of medical resources. On January 30, 2020, COVID-19 was declared as an international public health emergency by WHO due to the surge of cases across many countries (2).

The COVID-19 pandemic has substantially altered many aspects of our daily life. Researchers have studied COVID-19 impact on people's psychological health (3), perceived risk and public compliance (4), media communication (5), and government leadership (6). Besides the high infection rate, this pandemic also has a broad impact on the general population's health and well-being (7). Research found that the COVID-19 pandemic had a great impact on the medical system (8), which would inevitably affect the doctor–patient relationship (DPR). Healthy and positive DPR is crucial as it influences the quality of healthcare and empower the patient to cope with their illness during a pandemic (9). Patient's treatment compliance, satisfaction with the treatment procedure, and treatment outcome could also influence the DPR conversely (10–12). Moreover, DPR plays a role in the public's overall psychological health during COVID-19. A recent study reported that a high level of confidence in medical workers and satisfaction with health information are protective factors of the general population's mental and psychological health during the COVID-19 pandemic (13). Although DPR is crucial during usual medical consultation, few has investigated the DPR during the COVID-19 pandemic.

DPR may be influenced by many factors. Verbal and non-verbal communication, decision making, information sharing between patients and doctors, empathy, and doctors' medical knowledge are the factors that may affect the relationship between patients and doctors (14). During an emergency public health crisis such as the COVID-19 pandemic, the public's attitude toward doctors may change rapidly depending on how well medical professionals manage the disease and deal with their relationships with patients. The professionalism of the frontline medical workers reshaped the public's perception of Chinese doctors. Meanwhile, the media's reports and praise on frontline medical workers also enhanced the public's understanding and support of medical workers, which in turn could improve the DPR. However, due to social distancing limitations, doctors were forced to decrease face-to-face consultations with patients (15). These restrictions may lead to some miscommunication and misunderstanding between doctors and patients (16), which could deteriorate DPR. Poor DPR may lead to medical violence toward doctors, further reducing the doctors' working enthusiasm. Therefore, the purpose of our study was to provide a better understanding of DPR during the COVID-19 pandemic in China. We aimed to explore the following three questions:

1. To study the DPR status during the COVID-19 pandemic.
2. Is the doctor–patient trust perceived differently during the COVID-19 pandemic?
3. To study the predictor variables of the DPR.

Through this study, we hope to better understand the DPR in China during the COVID-19 pandemic and make some contribution in improving the DPR, as well as further healthcare and medical policy making.

## Methods

### Participants

This cross-sectional, retrospective, online survey was conducted between March 12, and March 30, 2020. The inclusion criteria were: (1) age 18 and above, (2) engaging in non-medical and health work, (3) living in China, (4) being able to read and write in Chinese, and (5) having medical treatment seeking experience during the pandemic (including online consultation). Participants' exclusion criteria were: (1) under 18 years of age, (2) illiterate, (3) cannot complete the questionnaire due to dementia or other severe cognitive dysfunction or severe mental disorder, and (4) had no experience of seeking medical help during the pandemic.

### Recruitment

Convenience and snowball sampling strategies were used to recruit participants in this study. Study flyer was posted on social media sites (e.g., WeChat, Weibo, QQ) and directed toward potential participants. The flyer explained the study purpose, and all participants could drop out of the survey at any time. Interested participants were given a link to the study's ethics approval consent form via a professional survey service—Questionnaire Star (https://www.wjx.cn). Participants who gave electronic informed consent were invited to complete demographic questionnaires and DPR measurements. This study was approved by the Ethics Committee of The Second Xiangya Hospital of Central South University (No. LYE2020041).

### Measures

#### Socio-demographics

Socio-demographic information of all participants included age, gender, education, monthly income, occupation, residency, medical expenses, and frequency of face-to-face medical consultation during the pandemic were collected.

#### General Trust in Medical Services

Participants' trust in medical services (self-perceived trust) before and after the COVID-19 outbreak was evaluated by asking “How much trust do you have in medical services before/after the coronavirus outbreak?” Participants' perceived attitudes on other people's trust in medical services (other-perceived trust) were measured by asking “How do you feel that most other people's trust in medical services before/after the coronavirus outbreak?” In total, four questions were asked; the ratings were made on a 5-point Likert scale, ranging from 1 (*very distrustful*) to 5 (*very trustful*).

#### Patient–Doctor Relationship Questionnaire (PDRQ-9)

The Patient–Doctor Relationship Questionnaire (PDRQ-9) is a 9-item questionnaire that evaluates the DPR in a primary care setting (17). It is rated on a 5-point Likert scale, ranging from 1 (*strongly disagree*) to 5 (*strongly agree*), with a total score ranging from 9 (very low quality) to 45 (very high quality). The questionnaire has been used in a previous Chinese sample with a Cronbach's alpha of 0.95 (18). Participants were asked to recall and rate the items before and during the COVID-19 pandemic.

#### Chinese Wake Forest Physician Trust Scale (C-WFPTS)

The Wake Forest Physician Trust Scale is an 11-item questionnaire that examines various dimensions of patients' trust in doctors (19). It was translated and validated into the Chinese version of the Wake Forest Physician Trust Scale (C-WFPTS) (20). This scale has two subscales: trust (7 items) and distrust (4 items, all coded reversely). In the validation sample, the C-WFPTS has Cronbach's alpha of 0.83. In this sample, we removed item 8, “I feel [my doctor] will release my personal information to unauthorized persons,” as the factor loading was low in the Chinese sample (21). The questionnaire is rated on a 5-point Likert scale, ranging from 1 (*strongly disagree*) to 5 (*strongly agree*). Participants were asked to rate the items once in this study.

#### Survey on Medical Violence Against Doctors

Participants' attitudes regarding medical violence against doctors were measured by asking two questions on a binary yes–no scale, which were “Have you ever participated in any verbal abuse against doctors?” and “Have you ever participated in any physical abuse against doctors?” Participants were asked to rate these items before and during the COVID-19 pandemic retrospectively. Participants were also asked how they perceived other people behave on these items before and during the COVID-19 pandemic. The change in medical violence was calculated by taking the difference between and before the COVID-19 pandemic and then factorized the change into four groups: *more violence, less violence, same violence*, and *no violence*.

#### Factors That Could Influence and Improve DPR

Participants were asked to pick five out of 12 or nine items that influence or improve DPR, respectively. Some examples of the items are “medical knowledge,” “communication,” “medical insurance,” “medical technology,” and “hospital management.” Details are shown in **Table 3**.

### Quality Control

To ensure the quality of data, not only do we have inclusion and exclusion criteria, but we also conducted quality control standards for this study to flag and exclude untrustworthy responses. First, questionnaires with multiple logic verification errors were eliminated. Second, participants could only answer once no matter which platform they use (i.e., computer, mobile phone). Third, participants who took <3 min to complete the survey were excluded. Finally, participants had to enter a verification code when submitting the response.

### Statistical Analysis

The change in general trust in medical services was analyzed using a repeated-measures analysis of variance (rm-ANOVA). Simple slope analysis was used to test the difference between self-perceived trust and other-perceived trust in medical services. rm-ANOVA and one-way ANOVA were used to analyze the relationship between eight participant demographics and DPR, and trust. Essentially, each demographic variable becomes an independent variable, while DPR and trust become the dependent variables. Additionally, Bonferroni correction, set at *p* < 0.00625, was used to correct for multiple testing for these eight ANOVAs. Descriptive statistics and chi-squared (χ^2^) tests were applied to measure the differences in medical violence against doctors between before and during the outbreak of COVID-19. One-way ANOVA was used to examine which medical violence factors were associated with changes in DPR, using changes in medical violence factors as the between-subjects variable and changes in DPR as the dependent variable. Cochran's Q was used to examine the factors that affect and improve DPR separately. All *post hoc* analyses were conducted using Tukey's test. Two stepwise linear regression analyses with all demographic variables as predictors were used to examine the relationship between DPR and trust. Demographics and DPR/trust scores were selected with a stepwise backward method. The statistical significance level was set at *p* < 0.05 (two-sided). All data analyses were conducted with R 4.0.3.

## Results

### Demographic Information

A total of 2,000 participants filled out the survey questionnaire; 52 were excluded as they did not complete the questionnaire, another 35 were excluded as their response could not be verified electronically (the survey platform has a strict logical verification rule), and 10 were excluded as the time of completion was less than the minimum time required to complete the survey (<3 min). Hence, the final sample consisted of 1,903 participants with a response rate of 95%. Among these samples, the average age was 35.78 years (*SD* = 10.51, range = 18–80 years old), 60.00% were female (*N* = 1,142), 1,412 had at least a college degree (74.20%), and 1,590 lived in city (83.60%). Additionally, 764 participants' medical expenses had a moderate impact on their family's economy (40.10%), 917 participants (48.20%) occasionally (1–2 times) visited the doctor, and 786 participants (41.30%) visited the doctor at the prefecture level. Other demographic characteristics are shown in Table 1.

**Table 1:** Sample characteristics and doctor–patient relationship variables.

**Variables**		***N* = 1,903**	**PDRQ-9**			**C-WFPTS**	
			**Pre-COVID-19 (SD)**	**COVID-19 (SD)**	***p*-value**	**Mean (SD)**	***p*-value**
Gender (%)	Female	1,142 (60.00)	34.60 (6.09)	37.57 (4.63)	N.S.	36.04 (4.43)	N.S.
	Male	761 (40.00)	34.96 (5.88)	37.77 (4.56)		36.28 (4.28)	
Age		35.78 (10.51)					
Education (%)	Below High School	144 (7.60)	35.15 (5.68)	38.03 (4.65)	N.S.	36.33 (5.22)	N.S.
	High School	347 (18.20)	34.74 (5.94)	37.70 (4.61)		36.41 (4.35)	
	College	1,181 (62.10)	34.86 (6.05)	37.63 (4.51)		36.06 (4.18)	
	Master's and above	231 (12.10)	34.31 (6.50)	37.39 (5.04)		35.97 (4.77)	
Monthly Income (%)	<50 k	771 (40.50)	34.87 (5.93)	37.72 (4.61)	N.S.	36.40 (4.57)	N.S.
	50–100 k	593 (31.20)	34.92 (5.87)	37.73 (4.53)		36.05 (4.34)	
	100–200 k	332 (17.40)	34.30 (6.21)	37.26 (4.74)		35.80 (4.13)	
	>200 k	207 (10.90)	34.76 (6.05)	37.75 (4.57)		35.92 (4.07)	
Occupation (%)	Civil servant	87 (4.60)	34.75 (6.32)	37.61 (5.02)	N.S.	36.36 (3.55)	*p* = 0.0025^**^
	Institution staff (schools, research, military, etc.)	649 (34.10)	34.47 (6.20)	37.39 (4.74)		36.00 (4.21)	
	Medical student	148 (7.80)	34.92 (6.14)	37.66 (4.75)		36.61 (4.72)	
	Non-medical student	127 (6.70)	35.33 (6.54)	37.96 (5.08)		37.15 (4.35)	
	Others	368 (19.30)	34.63 (5.20)	37.60 (4.16)		35.84 (4.72)	
	Retired	53 (2.80)	34.58 (5.93)	37.75 (4.37)		36.42 (4.60)	
	Self-employed	471 (24.80)	35.00 (6.11)	37.94 (4.51)		36.06 (4.29)	
Residency (%)	City	1,590 (83.60)	34.72 (6.01)	37.67 (4.54)	N.S.	36.11 (4.31)	N.S.
	Town	95 (5.00)	34.79 (4.74)	37.39 (3.89)		36.02 (3.95)	
	Village	218 (11.50)	34.85 (6.51)	37.59 (5.34)		36.36 (4.99)	
Medical Expenses (%)	Very little	89 (4.70)	35.74 (6.33)	38.33 (4.97)	N.S.	36.56 (4.36)	N.S.
	Little	299 (15.70)	34.97 (5.69)	37.93 (4.16)		36.54 (3.80)	
	Average	764 (40.10)	34.60 (6.01)	37.53 (4.64)		36.14 (4.33)	
	more than average	459 (24.10)	34.83 (5.64)	37.69 (4.23)		35.97 (4.10)	
	Huge	292 (15.30)	34.42 (6.74)	37.37 (5.33)		35.82 (5.34)	
Frequency of doctor visit (%)	Never	270 (14.20)	35.21 (6.37)	37.82 (5.00)	N.S.	36.46 (4.47)	*p* = 0.0028^**^
	Occasionally (1–2 times)	917 (48.20)	34.71 (6.07)	37.66 (4.67)		36.20 (4.44)	
	Sometimes (3–4 times)	463 (24.30)	34.59 (5.55)	37.60 (4.01)		35.92 (4.11)	
	Often (6–12 times)	210 (11.00)	34.25 (6.28)	37.22 (5.13)		35.58 (4.39)	
	Always (>12 times)	43 (2.30)	36.42 (5.47)	38.77 (3.80)		37.65 (4.63)	
Hospital level (%)	Individual clinics	82 (4.30)	34.38 (5.94)	37.59 (4.25)	N.S.	36.22 (4.20)	N.S.
	County	300 (15.80)	35.33 (5.67)	38.16 (4.25)		36.51 (4.39)	
	Township	130 (6.80)	34.62 (6.02)	37.35 (4.88)		36.12 (4.22)	
	Prefecture	786 (41.30)	34.62 (6.09)	37.47 (4.81)		36.06 (4.50)	
	Provincial and ministerial	584 (30.70)	34.69 (6.04)	37.71 (4.44)		36.03 (4.24)	
	Private	21 (1.10)	34.62 (7.16)	37.38 (5.63)		36.10 (4.79)	

*PDRQ-9 = Patient-Doctor Relationship Questionnaire. C-WFPTS = Chinese Wake Forest Physician Trust Scale*.

** *p < 0.00625*.

### DPR Before and During COVID-19 Measured by PDRQ-9

In this study, the Cronbach's alpha of PDRQ-9 was 0.90 for the pre-COVID-19 pandemic period and 0.85 for the COVID-19 pandemic period. rm-ANOVA revealed no significant effect of demographics or the interaction between demographics and time on DPR (*p* > 0.05). However, there was a main effect of time for all demographics after the Bonferroni correction, where DPR increased during the COVID-19 pandemic (*p* < 0.01). The mean PDRQ-9 score increased from 34.74 before the epidemic to 37.65 during the epidemic (Table 1).

### Trust Before and During COVID-19

**Figure 1:**
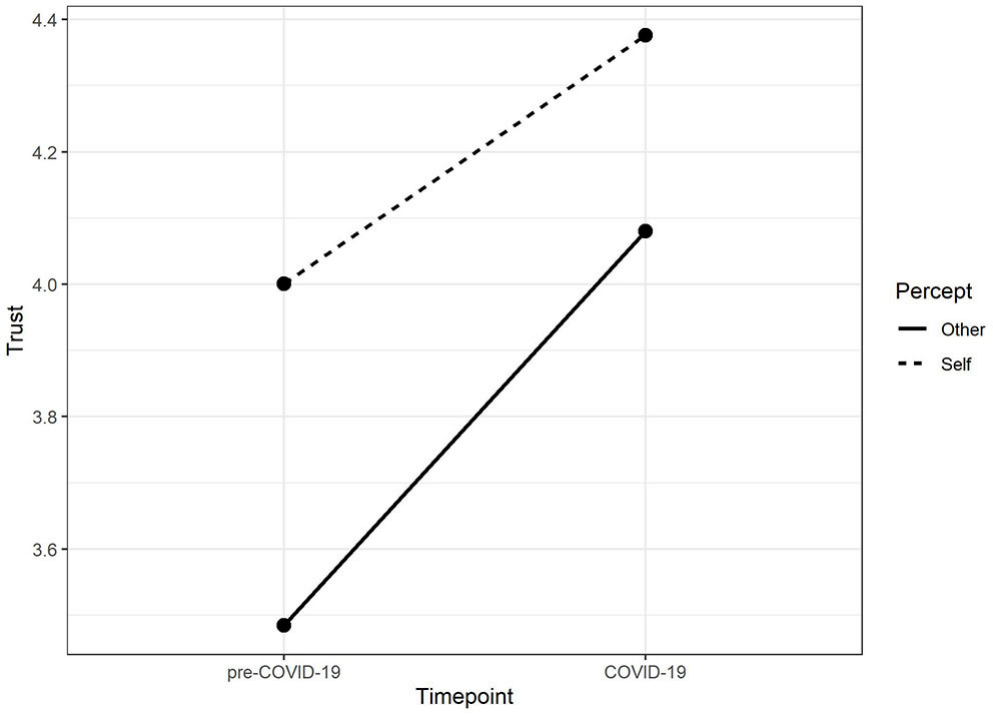
Change in perception of trust in medical services before and during COVID-19. Individuals perceived that their trust increased as well as that of others, and improvement of others was even greater during COVID-19.

The changes of general population's trust in medical services during the pandemic are shown in Figure 1. rm-ANOVA revealed that self-perceived trust increased significantly from 4.00 (*SD* = ±0.67) to 4.38 (±0.68; *p* < 0.001), and other-perceived trust increased significantly from 3.49 (±0.74) to 4.08 (±0.72; *p* < 0.001). *Post hoc* slope analysis revealed that the other-perceived trust increased greater than self-perceived trust (*p* < 0.001).

The average score of C-WFPTS reflecting patients' trust in doctors at the individual level during COVID-19 was at a high level (*M* = 36.13; *SD* = 4.37). In this sample, the Cronbach's alpha of the C-WFPTS was 0.68, the Cronbach's alpha of the trust subscale was 0.83, and the Cronbach's alpha of the distrust subscale was 0.29. Further correlation analysis revealed that the trust subscale was weakly and negatively correlated with the distrust subscale (*r* = −0.18, *p* < 0.001). Furthermore, the trust subscale was strongly and positively correlated with the total scale (*r* = 0.94, *p* < 0.001), and the distrust subscale was weakly and positively correlated with the full scale (*r* = 0.17, *p* < 0.001). As the distrust subscale had poor reliability in this sample, further analysis was conducted with only the trust subscale.

**Figure 2:**
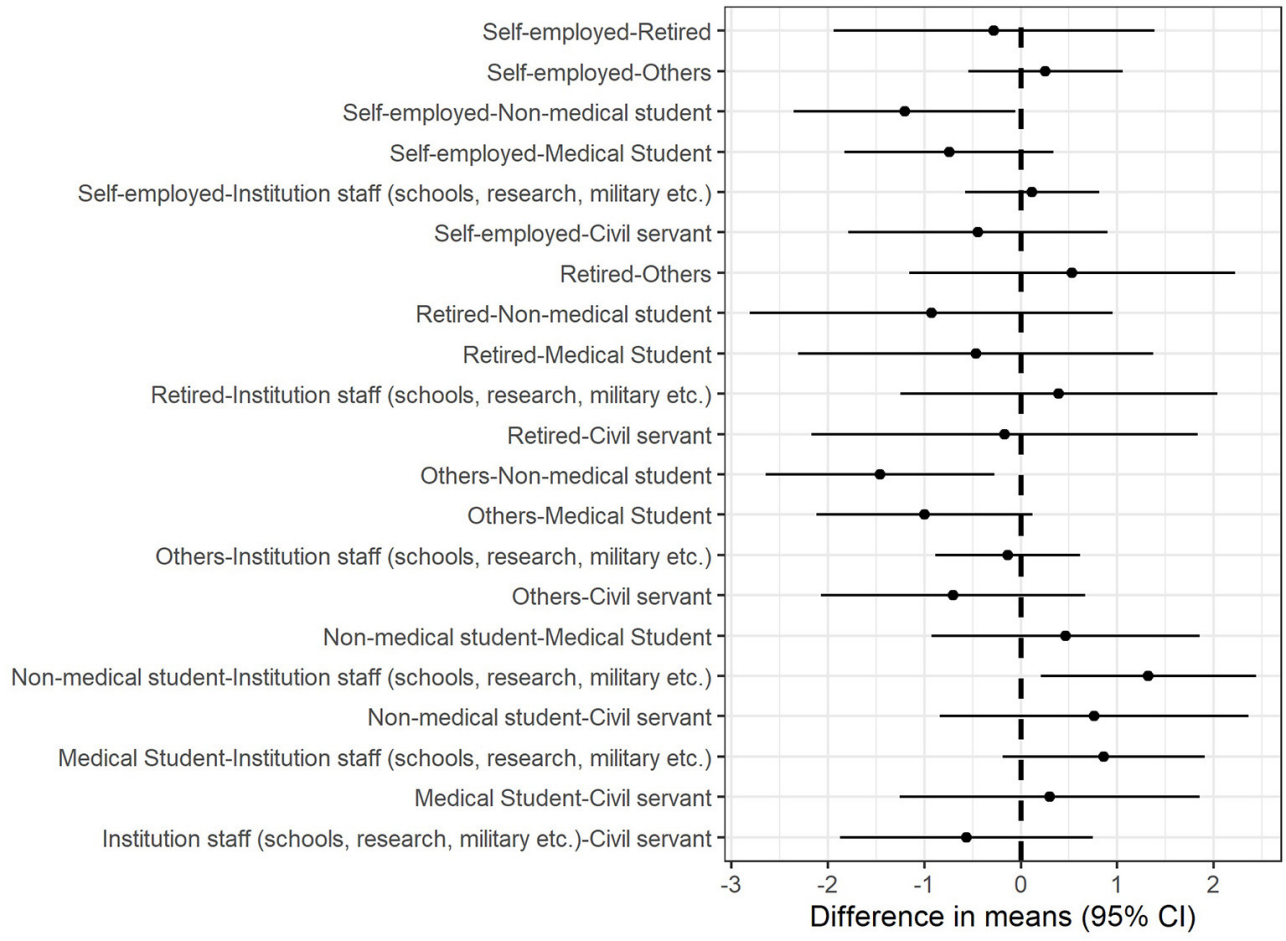
Marginal mean differences in trust for occupational groups. Self-employed persons, institutional staff, and other professionals had lower trust in doctors than non-medical students.

**Figure 3:**
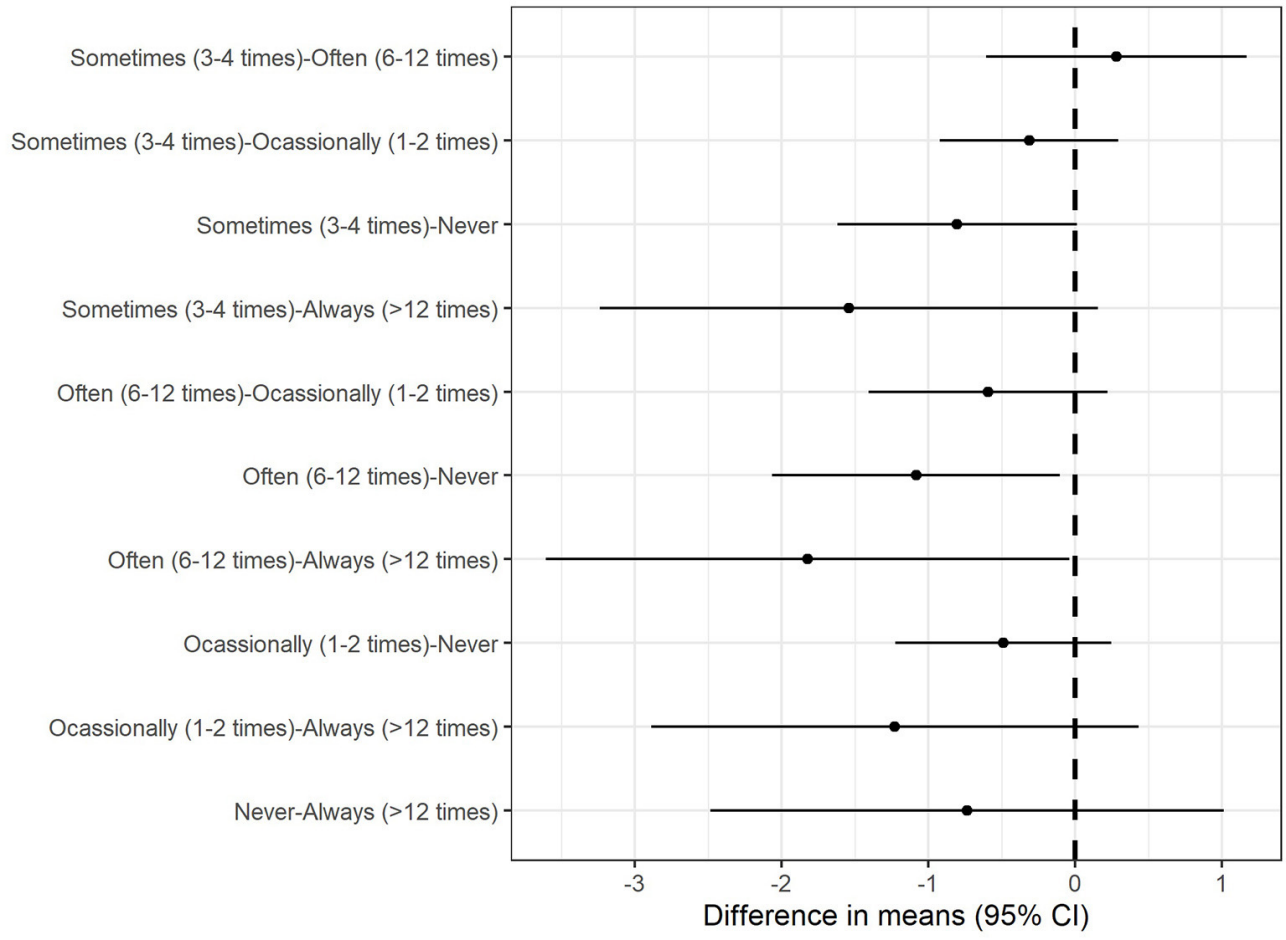
Marginal mean differences in trust for frequency of doctor visits. Individuals who had moderate face-to-face doctor–patient visits had lower trust in doctors than those who never or always visited the doctors face-to-face during the COVID-19.

ANOVA revealed that trust scores differed among occupations at *F*_(6, 1, 896)_ =3.39, *p* = 0.0025 (Figure 2), and frequency of doctor visits at *F*_(4, 1, 898)_ =4.05, *p* = 0.0028 (Figure 3). *Post hoc* Tukey's HSD test revealed that non-medical students (*M* = 37.15, *SD* = 4.35) had higher trust in doctors compared to self-employed (*M* = 36.06, *SD* = 4.29, *p* = 0.033), other professionals (*M* = 35.84, *SD* = 4.72, *p* = 0.0052), and institutional staff (*M* = 36.00, *SD* = 4.21, *p* = 0.0086). Individuals who often visited the doctor (*M* = 35.58, *SD* = 4.39) had lower trust in doctors than those who never (*M* = 36.46, *SD* = 4.47, *p* = 0.021) or always (*M* = 37.65, *SD* = 4.63) visited the doctor face-to-face during the COVID-19 (*p* = 0.042). There were no significant differences in other measures between other demographic variables for trust (Table 1).

### Medical Violence Against Doctors Before and During COVID-19

**Figure 4:**
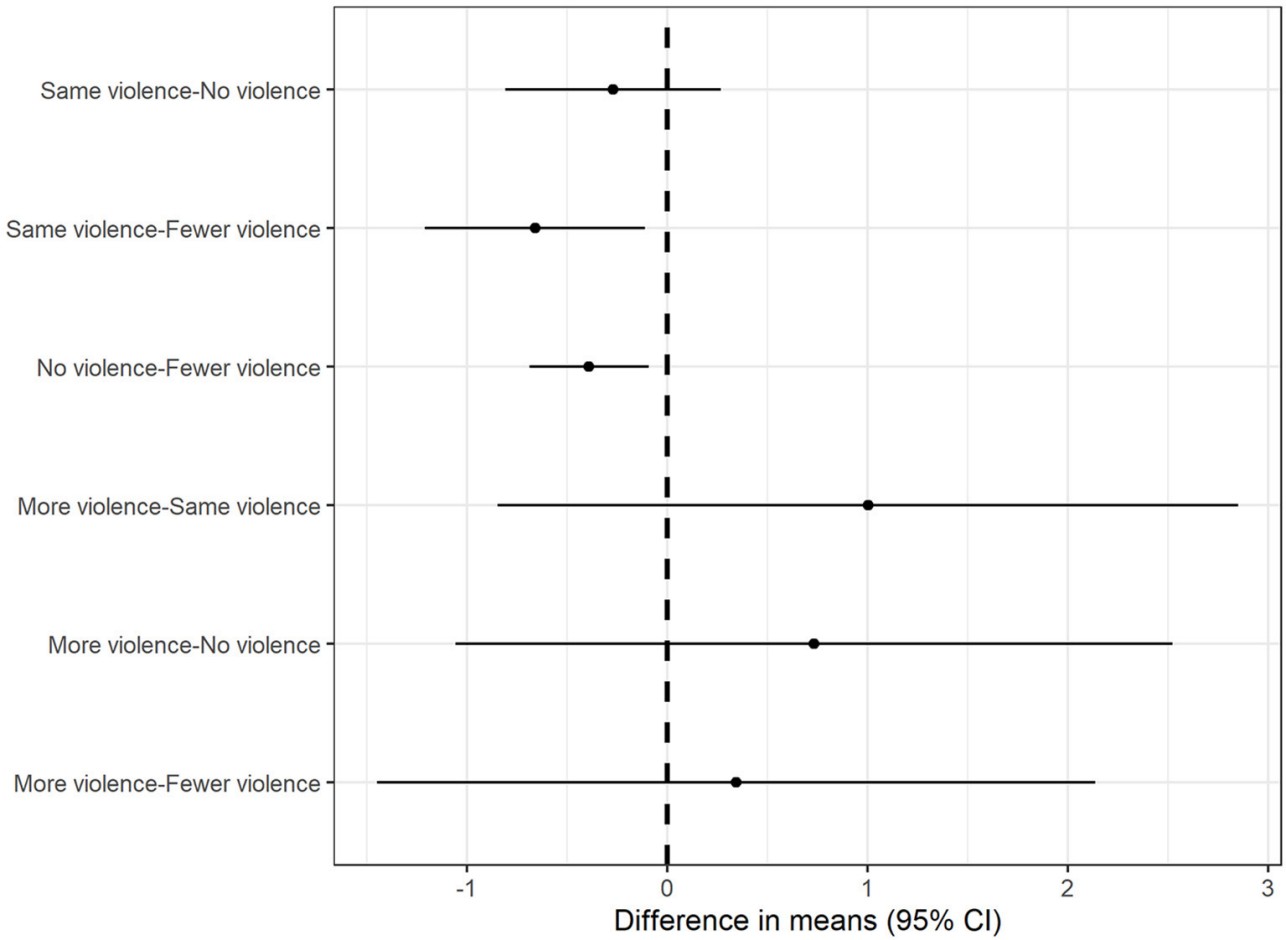
Marginal mean differences in DPR for verbal abuse. Individuals with fewer other-perceived verbal abuse had greater DPR score compared to individuals with either same or no verbal abuse.

Participants reported that during the COVID-19, medical violence against doctors by themselves and others decreased to approximately 20 percent before the outbreak. One-way ANOVA revealed that the change in other-perceived verbal abuse toward doctors significantly affected the change in DPR at *F*_(1, 1, 901)_ =12.63, *p* < 0.001. *Post hoc* Tukey's HSD test revealed that fewer violence than no violence (*p* = 0.0043) and the same violence (*p* = 0.011) positively changed DPR. There were no significant differences in any of the other variables. Medical violence against doctors before and during COVID-19 is shown in Figure 4.

### Factors That Affect and Improve DPR

Cochran's Q test revealed a significant difference among factors that affect DPR at *Q*_(11)_ = 4140.98, *p* < 0.001. The top five factors were difficulty in seeing a doctor and high consultation fees (72%), high expectations for doctors and perception that doctors know everything (60%), various reasons that lead to low trust between doctors and patients (57%), lack of knowledge from patients (54%), and poor communication between doctors and patients (41%).

Cochran's Q test revealed a significant difference among factors that improve DPR at *Q*_(8)_ = 3564.25, *p* < 0.001. The top five factors were improving communication between doctors and patients (73%), medical technology and services (67%), medical knowledge for patients (63%), medical legislations (59%), and medical insurance (59%). Details are shown in Table 2.

**Table 2:** Factors that affect and improve doctor–patient relationship.

**Factors**	**Description**	**Percentage**
Factors that affect DPR	Various reasons that lead to low trust between doctors and patients	57%
	It is difficult and expensive to see a doctor	72%
	The public's lack of knowledge about the disease, diagnosis, treatment process, and prognosis of the disease	54%
	Patients have high expectations of doctors, thinking that doctors know everything	60%
	Few doctors receive incentives and rebates, which affected the overall image of doctors	39%
	Negative or untrue reports of medical and pharmaceutical industries by the media	26%
	It is difficult to resolve medical disputes through formal channels, and the cost of medical disputes is too low, leading to a small number of people using medical disputes to seek benefits	30%
	The medical technology level and service quality are not high	13%
	Doctor–patient communication problems (the doctor is too busy or the communication is not in place, etc.)	41%
	Hospital management is not in place, and medical disputes are not handled in time	16%
	Low medical insurance reimbursement ratio	18%
	Others	2%
Factors that improve DPR	Extensively publicize medical science knowledge, so that the public understand that medicine is not a panacea	63%
	Improve medical technology and improve service quality	67%
	Improve communication between doctors and patients, such as reducing the intensity of medical and nursing work and allowing more time to serve patients	73%
	Improve media accountability, strengthen positive medical reports, and eliminate fake news	42%
	Improve the legal handling of medical disputes, promote legislation, and crack down on malicious medical disturbances	59%
	Improve the medical security system, and increase the coverage of medical insurance	59%
	Improve hospital management, and strengthen medical ethics and medical style	50%
	Establish a good image of medical staff	12%
	Others	2%

### Factors Predicting DPR and Trust

Backward stepwise linear regression for DPR revealed a significant model at *F*_(3, 4, 160)_ =279.80, *p* < 0.001, *R*^2^= 0.87. Older age (*B* = 0.009, *p* = 0.013), pre-COVID-19 DPR (*B* = 0.70, *p* < 0.001), and trust (*B* = 0.028, *p* = 0.0084) were associated with higher DPR. Backward stepwise linear regression for trust revealed a significant model at *F*_(12, 1, 890)_ =27.72, *p* < 0.001, *R*^2^ = 0.14. Compared to self-employed persons, non-medical students (*B* = 1.18, *p* = 0.0012) and medical students (*B* = 0.89, *p* = 0.0096) were positively associated with trust scores. Additionally, compared to individuals who never visited the doctor face-to-face (only visited virtually) or always visited the doctor face-to-face, individuals who visited the doctor sometimes (*B* = −0.75, *p* = 0.0074) or often (*B* = −0.92, *p* = 0.0074) were negatively associated with trust scores. Pre-COVID-19 (*B* = 0.13, *p* < 0.001) and COVID-19 (*B* = 0.14, *p* = 0.0050) DPR was positively associated with trust scores. Details are shown in Table 3.

**Table 3:** Regression weights of trust in doctors and the doctor–patient relationship.

**Variables**		**PDRQ-9 (B)**	**C-WFPTS (B)**
Occupation (%)	Civil servant	–	0.63^***^
	Institution staff (schools, research, military, etc.)	–	0.10
	Medical student	–	0.93
	Non-medical student	–	1.22^**^
	Others	–	−0.14^***^
	Retired	–	0.38
	Self-employed (Ref Group)		
Frequency of doctor visit (%)	Never (Ref Group)		
	Occasionally (1–2 times)	–	−0.49
	Sometimes (3–4 times)	–	−0.80^**^
	Often (6–12 times)	–	−0.95^**^
	Always (>12 times)	–	0.48
Age		0.0091^*^	–
Pre-COVID-19 DPR		0.71^***^	0.13^**^
COVID-19 DPR		–	0.14^**^
Trust		0.42^***^	–
Model *R^2^*		0.87^***^	0.14^***^

* *p < 0.05,*

** *p < 0.01,*

*** *p < 0.001*.

## Discussion

This cross-sectional, retrospective study explored the DPR and its related factors during the COVID-19 pandemic from the patients' perspective. We also evaluated participants' attitudes toward DPR before the pandemic by asking them to recall their experience. Our results showed that the PDRQ-9 score increased during the pandemic, which indicated that, compared to the time before the outbreak of COVID-19, DPR improved. Participants' C-WFPTS mean score was at a high level, which means participants' trust in doctors was high. We also found that participants' violence against doctors decreased during the COVID-19 pandemic. Furthermore, we also identified some factors that may contribute to DPR and trust, which may contribute to improving DPR in China.

Compared to the period before COVID-19, participants had a better perception of DPR, which was consistent with the main theme of doctor–patient interaction in mainstream media during the lockdown. Since no other quantitative research on DPR during the pandemic has been found, direct comparisons cannot be made. However, the average PDRQ-9 score in our study was significantly higher than that in previous similar studies using the same scale (22). During the 2003 SARS outbreak, it was also noted that the relationship between doctors and patients improved (23). Hence, we have reasons to believe that DPR during the COVID-19 pandemic improved compared to that before the COVID-19 pandemic. This improvement of DPR was the result of many factors, such as the government's public health strategies during the pandemic (24), professionalism shown by medical workers (25), and positive media reports (26). Our study also showed a significant positive correlation between trust and DPR. The more patients trust their doctors, the better their relationship will be, and vice versa. Trust is crucial in medical practice, as it is the basis and core of the harmonious DPR (27, 28) and the key to the effective operation of the medical system (29).

Another finding was that participants had a higher perception of trust in doctors and medical services. The mean value of trust in medical service during the pandemic was significantly higher than that before the pandemic and a national cross-sectional survey conducted in 2008 (30). During the COVID-19 pandemic, frontline medical workers' actions had received high praise from the Chinese public. Meanwhile, the stories of frontline medical workers' fighting with the disease had been widely shared on social media (31). This was consistent with other studies, as Sun et al. also found that good press coverage can increase the public's trust in medical workers, thereby improving the DPR (32). However, in recent years, the situation of doctor–patient trust in China is not optimistic. According to a survey conducted by China Youth Daily in 2013, about 70% of patients did not trust doctors (33). Our study also found that the important factors that affect DPR include doctor–patient distrust. Mistrust between medical workers and patients in China may be due to cultural reasons, imbalance of medical resources, privatization of medical services, lack of medical knowledge within patients, and so on (34).

In recent years, the Chinese DPR has escalated from simple distrust to a surge of conflict. Chinese doctors have been facing increasingly serious personal safety threats at work, including verbal and physical abuse, injury, and even murder (35–38). A representative study showed that of 1,656 doctors in Shanghai, Hubei, and Gansu, 92.75% reported that they had suffered verbal abuse, 88.1% had been threatened, and 81.04% had been physically assaulted (39). In our study, participants also reported physical and verbal abuse toward doctors either by themselves or by others during the pandemic. However, our study also confirmed that individuals who had reduced violence against doctors would have a positive attitude toward DPR. Hence, there is an urgent for health services to establish an environment of trust between medical workers and patients and reduce medical violence against medical workers in China.

Some previous studies attempted to explore potential measures to improve DPR. Our results indicated that higher doctor–patient trust would help improve DPR. Du et al. reported that doctor–patient communication, improving medical service quality, and service satisfaction are important issues in rebuilding doctor–patient trust (40). Nie et al. believed that medical professionalism plays an important role in rebuilding doctor–patient trust (41). Sun et al. claimed that regulating media reports can restore doctor–patient trust (32). In our study, participants believed that the five most important aspects for improving DPR are doctor–patient communication, medical technology and services, patient medical knowledge, medical legislation, and medical insurance, which were partly consistent with the previous studies. public policy makers could consider the mentioned factors that may affect DPR when making further strategies in order to improve DPR in the future.

Furthermore, our study found that, compared to other occupations, students were positively associated with trust. We ascribed this to students' predisposition, who mainly were younger individuals than those in other occupational groups. Other occupational groups may have more preconceptions of medical professionals from having more experiences as members of society. We also found that the number of face-to-face doctor–patient visits during the pandemic were related to trust, with moderate face-to-face visits related to lower trust rather than never (only visited virtually) or always visits. A reasonable explanation is that participants who frequently visited doctors may be more likely to form a longitudinal relationship with doctors, which could enhance trust. Similarly, some researchers found that a sustained relationship would likely increase trust and interpersonal relationships between doctors and patients (42). Amid the pandemic, virtual visits were encouraged due to the lockdown and physical distancing requirements, (43), it is not clear how much virtual visits would affect DPR and worth further study.

## Limitations

Nevertheless, some limitations need to be noted. First, the convenience sampling strategy and online survey method may lead to selection bias. The results of our study may not reflect the whole population's attitude. Second, this was a cross-sectional, retrospective study, in which participants' attitude of DPR before the pandemic may not be as accurate as real-time attitude due to memory bias. A larger sample, a longitudinal study should be considered in further research. Third, we did not collect further demographic information, such as the province where the participants lived. There may be differences between provinces on the perception of trust and DPR due to different cultural reasons. Future studies may examine these differences for policymakers to manage public health more strategically. Finally, this was a quantitative study, in which participants' attitude of DPR only reflects through the score of each scale. Qualitative study, such as in-depth interviews, can also help us understand DPR to some extent. Further study could consider combining a quantitative interview with a qualitative interview, as well as a longitudinal study to better understand DPR comprehensively.

## Conclusions

In conclusion, this study showed that the DPR and trust between patient and medical workers in China increased during the pandemic. Furthermore, our study identified some key factors that may affect or predict DPR and trust. The findings of this study help to understand DPR in China better, which may contribute to further medical policy making and physician practicing in order to improve DPR and doctor–patient trust.

## Data Availability Statement

The raw data supporting the conclusions of this article will be made available by the authors, without undue reservation.

## Ethics Statement

The studies involving human participants were reviewed and approved by Ethics Committee of The Second Xiangya Hospital of Central South University (No. LYE2020041). The patients/participants provided their written informed consent to participate in this study.

## Author Contributions

The study was conceptualized by TL, QW, SC, and YZ. The database was organized by YiW, ML, YuL, and YuW. Data analysis was done by YZ and WY. The manuscript with inputs was drafted by YZ, SC, and WY and reviewed by TL, YaL, SC, DW, YM, and XW. All authors read and approved the final manuscript.

## Conflict of Interest

The authors declare that the research was conducted in the absence of any commercial or financial relationships that could be construed as a potential conflict of interest.
